# Occurrence of polybrominated diphenyl ethers in floor and elevated surface house dust from Shanghai, China

**DOI:** 10.1007/s11356-018-1968-4

**Published:** 2018-04-24

**Authors:** Dong Niu, Yanling Qiu, Li Li, Yihui Zhou, Xinyu Du, Zhiliang Zhu, Ling Chen, Zhifen Lin

**Affiliations:** 1 Key laboratory of Yangtze River Water Environment, College of Environmental Science and Engineering, Tongji University, Shanghai, 200092 China; 2 State Key Laboratory of Pollution Control and Resource Reuse, College of Environmental Science and Engineering, Tongji University, Shanghai, 200092 China; 3 Shanghai Key Laboratory of Chemical Assessment and Sustainability, College of Environmental Science and Engineering, Tongji University, Shanghai, 200092 China

**Keywords:** Polybrominated diphenyl ethers (PBDEs), House dust, Floor dust (FD), Elevated surface dust (ESD), Temporal variation, Human exposure

## Abstract

House dust is the main source of human exposure to flame retardants by ingestion. This study investigated the occurrence of polybrominated diphenyl ethers (PBDEs) in indoor dust from 22 houses in Shanghai, China. House dust was separately collected from the floor and elevated furnishings surface (mostly between 0.5 and 2 m height) for comparison. The concentrations of ∑_22_ PBDEs ranged from 19.4 to 3280 ng/g (with a geometric mean of 203 ng/g) and from 55.1 to 792 ng/g (with a geometric mean of 166 ng/g) in floor dust (FD) and elevated surface dust (ESD), respectively. BDE-209 was the predominant congener, accounting for about 73.1% of total PBDE burdens. In terms of congener profiles, the comparison of FD and ESD revealed no significant differences except for the ratio of BDE-47/BDE-99. ESD samples displayed a ratio of BDE-47/BDE-99 very similar to commercial penta-BDE products DE-71 while the ratio in FD was exceptionally higher. Significant correlation was found between concentrations of commercial penta-BDE compositions in FD and ESD (*p* < 0.05). Except for some occasional values, PBDE levels in house dust exhibited temporal stability. Human exposure to PBDEs via dust ingestion was estimated. The highest daily intake of PBDEs was for toddlers by using 95th percentile concentrations of PBDEs via high dust ingestion in FD (23.07 ng/kg bw/day). About 20-fold difference in exposure estimates between toddlers and adults supports that toddlers are facing greater risk from indoor floor dust. Expectedly, this study highlighted the point that residents in Shanghai were exposed to low doses of PBDEs in house dust.

**Electronic supplementary material:**

The online version of this article (10.1007/s11356-018-1968-4) contains supplementary material, which is available to authorized users.

## Introduction

Polybrominated diphenyl ethers (PBDEs) are a group of anthropogenic organic compounds, which have been widely used as additive flame retardants to slow the spread of fire in many consumer products such as insulation building materials, electrical and electronic goods, textiles, and furniture since 1970s (Hassan and Shoeib 2015). PBDEs were originally produced in three commercial products: penta-, octa-, and deca-BDE products (Wilford et al. 2005). PBDEs may enter the environment through volatilization from various products containing PBDEs, which are thought to be lipophilic, bioaccumulative, degradation-resistant, and toxic (Legler and Brouwer 2003; Harrad and Diamond 2006; Kicinski et al. 2012). PBDEs have been reported to be endocrine disruptors and cause neurohehavioral dysfunctions in animal and human studies (Meeker et al. 2009; Herbstman et al. 2010; Bramwell et al. 2016). In response, production of penta-BDEs and octa-BDEs was firstly prohibited and later listed as persistent organic pollutants (POPs) in 2009 (UNEP 2009). Further, deca-BDE was added to the annexes A for elimination in 2017 (UNEP 2017). As consumer products containing large amounts of PBDEs are still in use, the research on PBDEs will continue to be a hot topic in the next years.

Indoor microenvironment, where people spend over 80% time in their lives (Johnson-Restrepo and Kannan 2009), represents a main source of flame retardants. Ingestion is identified as a major pathway of human exposure to PBDEs especially for toddlers (Jones-Otazo et al. 2005). Toddlers may have greater PBDE body burdens than adults, which is relevant to frequent hand-to-mouth behaviors on the floor, whereas adults have more contacts with elevated surface of furnishings. Moreover, there is no consolidated standard method for sampling indoor dust to assess human exposure risk of PBDEs. Without considering the potential influence of dust type or sampling elevation, most studies usually collect mixed house dust (mostly floor dust) by vacuum cleaners to roughly represent the whole house dust (Thuresson et al. 2012; Hoffman et al. 2015; Coelho et al. 2016; He et al. 2017). The limitation of active sampling is that this method may not reflect the actual human exposure to PBDEs in house dust, while passive sampling method may be preferable. To date, few studies compared the levels of PBDEs in indoor dust collected at different heights. PBDEs in house dust of different heights may have different levels and fates, which probably have implications for improved understanding of potential health impacts of PBDEs. In a research of a California campus, Allgood et al. (2017) suggested higher PBDE levels of exposure through elevated surface dust (ESD) than those through floor dust (FD). Al-Omran and Harrad (2016) found that concentrations of ∑tri-hepta-BDEs (the sum of tri-BDEs to hepta-BDEs) and BDE-209 in ESD differed from those in FD (*p* < 0.05). Levels of eight PBDE congeners including BDE-209 in ESD from 17 elementary schools of South Korea were lower than those in FD (Wu et al. 2010). Therefore, it is necessary to compare PBDEs in house dust of different heights, and assess human exposure risks of PBDEs for both toddlers and adults. In addition, temporal variation may influence the levels and fates of PBDEs in indoor dust and hence influence the human exposure. Up to now, several investigations of temporal variation of PBDEs in indoor dust have been carried out (Allen et al. 2008; Batterman et al. 2009; Muenhor and Harrad 2012; Yu et al. 2012; Cao et al. 2014b). However, no significant trends were corroborated owing to comparatively short sampling period and limited samples.

In this study, 22 PBDE congeners were analyzed in floor dust and elevated surface dust collected from Shanghai during the year of 2016. The objectives of this study were to (1) identify the PBDE levels and composition in house dust from Shanghai, (2) compare the difference of PBDE congener profiles between floor dust and elevated surface dust, (3) reveal the temporal variation of PBDEs in floor dust during the whole year, and (4) evaluate human exposure via dust ingestion.

## Materials and methods

### Dust sampling

In the whole year of 2016, FD samples were collected from 22 representative houses every second month in Shanghai. Detailed information about house conditions is provided in the supplementary material. In each house, five FD samples were collected separately from the living room, washing room, kitchen, and two bedrooms by using pre-cleaned brushes at the same time. The participants were required not to clean the floor for 1 week before collecting FD samples. Elevated surface dust (ESD) samples were collected in every house by naturally falling into a 20 × 20 cm salver covered by aluminum foil. These salvers were placed on the surface of shelves, tables, or other furnishings (mostly between 0.5 and 2 m height). Plastic materials were avoided throughout the whole sampling procedures. In order to guarantee sufficient dust mass for analysis, five FD samples from each house were intentionally mixed together as one typical sample, while ESD samples were collected once in 1-year period. The total amounts of FD and ESD samples selected for analysis were 154 (*n* = 132 for FD, *n* = 22 for ESD). All dust samples were wrapped in solvent-cleaned aluminum foil and then sealed in polyethylene bags before taken back to the laboratory.

### Chemicals

Two standard mixtures of PBDEs, BDE-MXF (BDE-28, -47, -66, -85, -99, -100, -153, -154, -183) and BDE-OND (BDE-194, -195, -196, -197, -198, -199, -200, -201, -202, -203, -204, -205, -206, -207, -208, -209), were purchased from Wellington Laboratories Inc. (Guelph, Ontario, Canada). Individual standards of BDE-118 and BDE-138 were acquired from AccuStandard Inc. (New haven, CT, USA). All organic solvents used were of pesticide analysis grade. Silica gel (100–200 mesh) was acquired from Merck (Darmstadt, Germany).

### Dust sample preparation and extraction

In order to remove coarse particles and other residues (e.g., hair, plastics, floccule), bulk dust was sieved with a pre-cleaned and dried stainless steel sieve with 500 μm diameter of mesh. The sieved dust samples were homogenized thoroughly and stored in aluminum foil at − 20 °C. Extraction and cleanup procedures followed the same method as reported elsewhere (Zhou et al. 2016) with minor modifications. Briefly, each accurately weighed sample, typically 150 mg, was spiked with BDE-138 (0.05 ng/μL, 20 μL) as surrogate standard, and then extracted with 25 mL *n*-hexane/acetone mixture (1:1, *v*/*v*). Each sample was vortexed for about 1 min before sonication for 20 min. The extraction process was repeated two times and then extracts were evaporated by a rotary vacuum evaporator (R-210/215, BUCHI Labortechnik). Concentrated sulfuric acid (98%) was added for each sample to remove lipids and organic matters, before the supernatant was further concentrated to about 1 mL purging by a gentle nitrogen stream. The cleanup step was carried out using a Pasteur pipette packed with activated silica gel (0.1 g) and activated silica gel (0.9 g) impregnated with 98% concentrated sulfuric acid (2:1 *w*/*w*) from bottom to top. Columns were firstly rinsed with *n*-hexane, then the extracts were added and analytes were eluted with a mixture of n-hexane/dichloromethane (10 mL, 1:1 *v*/*v*). Prior to instrumental analysis, the collected extracts were blown down to incipient dryness under a gentle stream of nitrogen and then resolubilized in 200 μL of *n*-hexane. BDE-118 (0.05 ng/μL, 20 μL) was added as volumetric standard.

### Instrumental analysis

Twenty-two PBDE congeners were analyzed by Agilent 7890A gas chromatography coupled to a 5975C mass spectrometry (GC-MS) using negative chemical ionization (NCI) in the selected ion monitoring (SIM) mode and were identified by retention time using authentic reference standards. BDE-209 was quantified by monitoring bromide ion fragments (*m*/*z* 486.6 and 488.6) while the ions at *m*/*z* 79 and 81 were selected for other target compounds. Automated 1 μL injections with a CTC GC Pal autosampler were conducted on a Varian 450-GC connected to a Varian 320-MS. A programmable temperature vaporizing (PTV) injector was used with a DB-5MS capillary column (15 m × 0.25 mm i.d. × 0.10 μm film thickness; Agilent J&W) achieving the chromatographic separation. Helium was used as carrier gas at a set constant flow of 1.5 mL/min while methane as reagent gas. The ion source, quadrupole, and transfer line temperatures were set at 250, 150, and 290 °C, respectively. The GC oven was programmed as follows: 80 °C for 1 min, 15 °C/min to 300 °C, 2 °C/min to 310 °C and held for 5 min. The PTV injector temperature was set at 280 °C in a splitless mode for 1 min.

### Quality assurance/quality control

Prior to use, all laboratory glassware were baked out at 400 °C for 5 h and solvent-rinsed. Silica gel (100–200 mesh) was activated at 300 °C for 12 h. In order to assess potential contamination, one procedural blank was run in parallel with each batch of 8–10 samples. To guarantee repeatability, duplicate samples (every tenth sample) were analyzed, which showed the relative percent difference of less that 20% between samples and duplicates. For quantification of PBDE congeners, seven-point calibration solutions were analyzed to obtain the calibration curves with reliable regression coefficients of over 0.996. The limit of detection (LOD) was calculated as a signal to noise ratio (S/N) of three times while the limit of quantification (LOQ) was set to S/*N* = 10 or three times the mean value of the blank samples. More information on LOQ is provided in Supplementary Material (Table S1). The recovery (mean ± standard deviation) of surrogate standard was 92 ± 8.9% for BDE-138 and the reported concentrations in this study were not corrected with surrogate recovery.

### Statistical analysis

Data about the general statistical description on the concentrations of PBDEs in FD and ESD samples were calculated through the Microsoft Office Excel 2011. Shapiro-Wilk normality test was performed by GraphPad Prism 6 (GraphPad Software, La Jolla, USA) to confirm our data with a positively skewed distribution. Geometric mean (GM), geometric standard deviation (GSD), median, arithmetic mean (average), range, and logarithm were used to compare the data with those reported elsewhere. A value of 1/2 LOQ was applied in statistical analyses as concentrations for congeners that were below the LOD. Statistical significance was defined if a null hypothesis could be rejected at *p* < 0.05. Paired-samples *T* test was used to analyze the difference between FD and ESD. Pearson correlation coefficients were calculated to investigate the relationships of PBDE concentrations between FD and ESD.

## Results and discussion

### PBDE concentrations in FD and ESD

Table 1 presents the concentrations of 22 PBDE congeners measured in 132 FD and 22 ESD samples. Among the congeners, BDE-183, -201, -206, -207, -208, -209 were detected in all samples, while detection frequency of other PBDE congeners fell between 21.6 and 99.2% (Table S1). BDE-85 had the lowest detection frequency (21.6%) in house dust, which was similar to the data given by Frederiksen et al. (2009). The total concentrations of 22 PBDE congeners (∑_22_ PBDEs) varied from 19.4 to 3280 ng/g and from 55.1 to 792 ng/g in FD and ESD, respectively. The GM concentration of ∑_22_ PBDEs in FD (203 ng/g) was comparatively higher than that in ESD (166 ng/g); however, no significant difference was found (*p* < 0.05). BDE-209 was the most abundant congener, contributing 73.4 and 54.9% in FD and ESD, respectively. The levels of BDE-209 ranged from 11.8 to 3060 ng/g in FD, while in ESD the levels varied from 28.1 to 634 ng/g. It can be deduced that the concentration range of BDE-209 in FD (11.8–3060 ng/g) was much more scattered than that in ESD (28.1–634 ng/g). However, the difference on GM concentrations of BDE-209 in two groups was not significantly distinct (FD 160 ng/g; ESD 123 ng/g). In FD, GM concentrations of ten studied PBDE congeners were relatively higher and they were ranked as BDE-209 (160, ng/g) ≫ BDE-206 (12.7) ≈ BDE-207 (12.2) > BDE-208 (6.65) > BDE-196 (2.26) > BDE-194 (1.29) ≈ BDE-183 (1.45) ≈ BDE-205 (1.11) ≈ BDE-201 (1.09) > BDE-47 (0.65), while in ESD the similar trend was found as BDE-209 (123, ng/g) > BDE-207 (13.7) ≈ BDE-206 (11.2) > BDE-208 (7.35) > BDE-196 (1.61) = BDE-205 (1.61) > BDE-201 (1.09) ≈ BDE-99 (0.96) = BDE-197 (0.96) > BDE-47 (0.53). The dominant prevalence of BDE-209 in house dust is probably due to massive use of products containing BDE-209 and its low volatility, which makes it more liable to be found on particulates at room temperature (Shoeib et al. 2004). Except deca-BDE (BDE-209), concentrations of the total nona-BDEs (BDE-206, -207, -208) were 31.8 ng/g in FD and 33.1 ng/g in ESD (Table 1; Fig. S1), respectively, indicating the predominance of higher brominated BDE congeners in both FD and ESD. Total concentrations of nona-BDEs both exhibited a linear correlation with those of deca-BDE in FD (*R*^2^ = 0.25; *p* < 0.01) and in ESD (*R*^2^ = 0.74; *p* < 0.01).

**Table 1:** Concentrations (ng/g) of PBDE congeners in FD and ESD

Congener	Floor dust (*n* = 132)				Elevated surface dust (*n* = 22)			
	Average^a^	GM (GSD)^b^	Median	Range	Average	GM (GSD)	Median	Range
BDE-28	0.50	0.19 (0.02)	0.18	ND^c^-10.8	0.29	0.10 (0.01)	0.21	ND-1.12
BDE-47	1.79	0.65 (0.07)	0.48	ND-45.7	1.28	0.53 (0.09)	0.57	ND-7.42
BDE-66	0.53	0.21 (0.01)	0.18	ND-11.0	0.43	0.23 (0.01)	0.25	ND-1.37
BDE-100	0.39	0.10 (0.01)	0.13	ND-5.80	0.46	0.15 (0.01)	0.21	ND-2.50
BDE-99	0.98	0.41 (0.03)	0.39	ND-10.7	1.68	0.96 (0.04)	0.91	0.23–9.92
BDE-85	0.11	0.08 (0.01)	0.06	ND-1.38	0.43	0.21 (0.02)	0.16	ND-1.41
BDE-154	0.27	0.13 (0.01)	0.13	ND-1.73	0.25	0.1 (0.01)	0.15	ND-0.98
BDE-153	0.22	0.04 (0.01)	0.01	ND-2.39	0.04	0.01 (0.001)	0.01	ND-0.51
BDE-183	2.89	1.45 (0.25)	1.34	0.13–39.4	0.74	0.53 (0.06)	0.82	ND-1.17
∑tri-hepta-BDEs^d^	7.69	4.85 (0.43)	4.15	1.08–73.5	5.60	4.22 (0.23)	2.96	1.93–19.9
BDE-202	1.23	0.63 (0.04)	0.63	ND-18.2	0.81	0.68 (0.05)	0.80	0.21–2.25
BDE-201	2.33	1.09 (0.13)	1.04	0.06–46.1	1.35	1.09 (0.09)	1.14	0.21–3.37
BDE-204	0.30	0.17 (0.01)	0.13	ND-3.22	0.31	0.19 (0.01)	0.21	ND-1.12
BDE-197	2.49	1.19 (0.24)	1.16	ND-40.7	1.16	0.96 (0.17)	0.89	0.21–3.37
BDE-198, -199, -200, -203^e^	1.81	0.65 (0.08)	0.62	ND-44.0	0.73	0.40 (0.03)	0.65	ND-2.04
BDE-196	6.11	2.26 (0.91)	2.09	ND-150	2.07	1.61 (0.13)	1.56	ND-6.87
BDE-205	1.91	1.11 (0.12)	1.02	ND-13.3	2.03	1.61 (0.07)	1.52	0.41–5.62
BDE-194	3.68	1.29 (0.07)	1.12	ND-97.8	1.15	0.59 (0.03)	0.80	ND-4.33
BDE-195	1.76	0.29 (0.03)	0.25	ND-103	0.35	0.22 (0.01)	0.21	ND-0.35
∑octa-BDEs^f^	21.6	9.84 (0.76)	8.71	1.03–429	9.96	8.31 (0.64)	8.38	2.13–22.5
BDE-208	16.4	6.65 (0.53)	5.12	0.51–272	9.79	7.35 (0.35)	7.41	1.87–33.7
BDE-207	28.9	12.2 (1.11)	9.63	0.84–442	18.9	13.9 (1.23)	13.0	3.32–65.2
BDE-206	34.3	12.7 (1.15)	10.7	1.53–702	15.2	11.2 (1.09)	11.0	2.90–57.8
∑nona-BDEs^g^	79.6	31.8 (3.42)	27.8	3.10–1420	43.9	33.1 (1.33)	30.9	8.09–129
BDE-209	321	160 (7.34)	139	11.79–3060	170	123 (6.21)	160	28.1–634
∑_21_ PBDEs^h^	109	43.4 (4.52)	36.5	6.10–1890	59.4	42.7 (2.12)	42.5	12.4–159
∑_22_ PBDEs^i^	430	203 (8.61)	187	19.4–3280	229	166 (5.37)	225	55.1–792

^a^*Average* arithmetic mean

^b^*GM* geometric mean, *GSD* geometric standard deviation

^c^Not detected

^d^Sum of BDE-28, -47, -66, -85, -99, -100, -153, -154, -183

^e^Congeners are listed together due to co-elution in GC-MS analysis

^f^Sum of BDE-194, -195, -196, -197, -201, -202, -203, -204, -205

^g^Sum of BDE-206, -207, -208

^h^Sum of PBDEs without BDE-209

^i^Sum of PBDEs including BDE-209

The concentrations of PBDEs in house dust measured in different countries varied in almost three orders of magnitude (Table S2), which was possibly due to different fire safety standards and living habits. As variances in sampling period and procedure among studies, it is difficult to make an accurate comparison for total PBDEs. Considering that BDE-209 was the dominant congener of indoor dust samples in almost all researches, the level of BDE-209 could be used for literature comparison. Concentrations of BDE-209 in the UK and USA were extremely higher than those in other countries, possibly owing to large amounts of products containing PBDEs still in use and different fire safety standards in these two countries. In detail, median concentration of BDE-209 in this study (139 ng/g for FD) was similar to the concentration in Turkey (138 ng/g) (Civan and Kara 2016), lower than that of Poland (270 ng/g) (Korcz et al. 2017) and Portugal (270 ng/g) (Coelho et al. 2016), and only one-tenth of that in the USA (1720 ng/g) (Stapleton et al. 2014) and UK (2660 ng/g) (Al-Omran and Harrad 2016). With respect to other PBDE congeners, levels of penta- and octa-BDEs were much lower than those in European countries like Turkey. Civan and Kara (2016) reported that the concentration of total penta-BDEs in 40 house dust samples of Turkey (136 ng/g) was almost two orders of magnitude higher than that in this study. The levels of BDE-183 and total octa-BDEs were 20 times and 4 times higher than those in this study, respectively. To some extent, it reflects a significant difference of penta-BDE and octa-BDE products used in China and Turkey, regardless of the ban of commercial BDE products all over the world. In a previous study, Xu et al. (2016) reported that the mean concentration of BDE-209 in household dust from Shanghai (*n* = 8) was 499 ng/g, which was very similar to the result in this study. Concentrations of main PBDE congeners such as BDE-47, BDE-99, BDE-183, and BDE-209 in the dust from Hangzhou (Sun et al. 2016), Nanjing (Wang et al. 2015), and Shanghai (Xu et al. 2016) were in the same order of magnitude, which indicates that there were to some extent geographical similarities of PBDEs in house dust from Yangtze River Delta. While comparing levels of BDE-209 in Shanghai from this study with those in 23 provinces across China (not including Shanghai), we found that GM concentrations of BDE-209 in this study were approximately 5 times lower than those in other cities of China (Zhu et al. 2015). Different levels of economic development and living habits may lead to the discrepancy of PBDE levels in different parts of China. Moreover, it is noteworthy that discontinuous dust sampling sizes and sampling density in these investigations also influence the levels of PBDEs in house dust (Cao et al. 2014a).

To sum up, the concentrations of PBDEs in this study were in a relatively lower level compared with European and North American countries and regions except for some regions in Africa (Hassan and Shoeib 2015) and Asia (Khan et al. 2016).

### Congener profiles

The predominant PBDE congener detected in this study was deca-BDE, accounting for 41.4–93.3% of the total PBDEs burden with an average of 73.1% (Fig. S2). As the secondarily abundant homolog, nona-BDEs (BDE-206, -207, -208) contributed 6.3–36.4% of the total PBDE burden except that they contributed over 40% in three samples of one apartment. Lower brominated PBDE congeners (tri-BDEs to hepta-BDEs) were of small proportion probably owing to the phase-out of commercial penta- and octa-BDE products in 2009. To illustrate, penta-BDEs were present in a very low proportion of less than 2% except that they contributed over 3% in only six samples. Generally, this was consistent with the consumption profile of commercial PBDE mixtures in China, where deca-BDE mixture was the most widely used in the past decade (Yu et al. 2012; Cao et al. 2014a; Zhu et al. 2015). Deca-BDE commercial mixture, such as Saytex 102E and Bromkal 82-0DE, comprises more than 97% BDE-209 (Schecter et al. 2005). It was thought reasonable that the proportion of BDE-209 in house dust was smaller than that in commercial products due to the existing commercial penta-BDE and octa-BDE products. The ratio of ∑nona-BDEs/∑deca-BDE was in the range of 8.09–40.5% (median 20.7%) in this study, which far surpassed that in commercial deca-BDE products (3.09%) (Wilford et al. 2005). Various proportions of BDE-209 in two groups of dust samples may reveal different usage of electronic products within houses (i.e., different types of computers, ventilation equipment, and plastic casings for electrical appliances) (Stapleton et al. 2005). From another perspective, photolytic or thermal degradation of BDE-209 into lower brominated PBDE congeners (mainly nona-BDEs) resulted in disparate proportions of BDE-209 in house dust (Stapleton and Dodder 2008). Wang et al. (2010) also reported the presence of BDE-202, as well as the BDE-197/ BDE-201 and the nona-BDEs/deca-BDE ratios in dust samples were probably indicative of environmental degradation of deca-BDE.

### Comparison and relationship between FD and ESD

PBDE concentrations of houses (*n* = 22) with both FD and ESD were log-transformed, and paired-samples *T* tests were applied to test whether concentrations of 22 PBDE congeners in FD would exceed those in ESD samples. The results showed that concentrations of BDE-85, BDE-153, BDE-183, and BDE-209 in FD exceeded significantly those in ESD, with (*p* < 0.05) 0.0106, 0.0007, 0.0064, and 0.0440 respectively (Table S3). Similar results were also found from those in elementary schools of South Korea (Wu et al. 2010), whereas opposite conclusions were in America and Norway indoor dust (Cequier et al. 2014; Allgood et al. 2017). One possible explanation for our results is that the majority of floating dust particles, which PBDEs adhered to in the air, eventually drifted down and accumulated on the floor. Another possibility is that PBDE concentrations were negatively correlated with surface loadings (ng dust per m^2^ of floor area) (Harrad et al. 2008). In this study, ESD samples were collected in much smaller areas (0.04 m^2^ salver) while FD samples were collected in every individual room floor (typically 10–30 m^2^). Accordingly, relative higher dust loadings in ESD samples resulted in lower levels of PBDEs. Harrad et al. (2008) hypothesized that “dilution” occurs at higher dust loadings, which was in line with our assumption.

In commercial penta-BDE mixtures such as DE-71 and 70-5DE, the ratios of BDE-47/BDE-99 were approximately 0.6 and 0.8, respectively (Yu et al. 2012), whereas they were 1.58 (FD) and 0.55 (ESD) from this study (*p* < 0.05). The concentrations of BDE-99 in ESD were one- to twofold higher than those in FD, while the BDE-47 level in ESD was moderate to that in FD. In this regard, ESD samples displayed a ratio very similar to commercial penta-BDE mixture DE-71, whereas FD samples displayed an aberrant ratio compared with commercial products. In a previous study, Stapleton et al. (2005) found that the removal of one of the bromine atoms from BDE-99 resulted in the formation of BDE-47 and debromination of BDE-99 could occur in house dust. So it could be hypothesized that debromination from higher brominated congeners to BDE-47 in FD leads to higher ratio of BDE-47/BDE-99. It seems like more formation of stable BDE-47 occurred in FD, but there is not yet a clear-cut explanation of higher rate of BDE-47/BDE-99 in FD samples and more data is required to fully evaluate the hypothesis.

In this study, a specific correlation matrix for houses (*n* = 22) with both FD and ESD samples was compiled to examine relationships of three commercial BDE compositions between in FD and in ESD. Scatterplots and Pearson correlation coefficients comparing PBDEs concentrations in FD and ESD are presented in Fig. S3. The significant linear relationship observed between two groups of house dust was for penta-BDEs (*R*^2^ = 0.89, *p* < 0.0001), which indicated that penta-BDEs in FD and ESD might be derived from the same source. The stability of penta-BDEs in FD and ESD was another possible explanation for this significant linear relationship. Octa-BDEs in two groups were linearly correlated with each other (*R*^2^ = 0.22, *p* < 0.05), while the correlation for deca-BDE was not significant (*R*^2^ = 0.20, *p* > 0.05). Deca-BDE may derive from various consumer products containing PBDEs or emerging brominated flame retardants. On the other hand, the debromination of BDE-209 into lower brominated congeners was probably spatially different. Deca-BDE demonstrated positively significant relationship with octa-BDEs in each group, which was in accordance with another study (Batterman et al. 2009). However, no distinct associations were found between penta-BDEs and octa-BDEs in ESD (*R*^2^ = 0.0036, *p* > 0.05), as well as penta-BDEs and deca-BDEs in ESD (*R*^2^ = 0.0025, *p* > 0.05).

### Temporal variation of PBDEs in floor dust

**Fig. 1:**
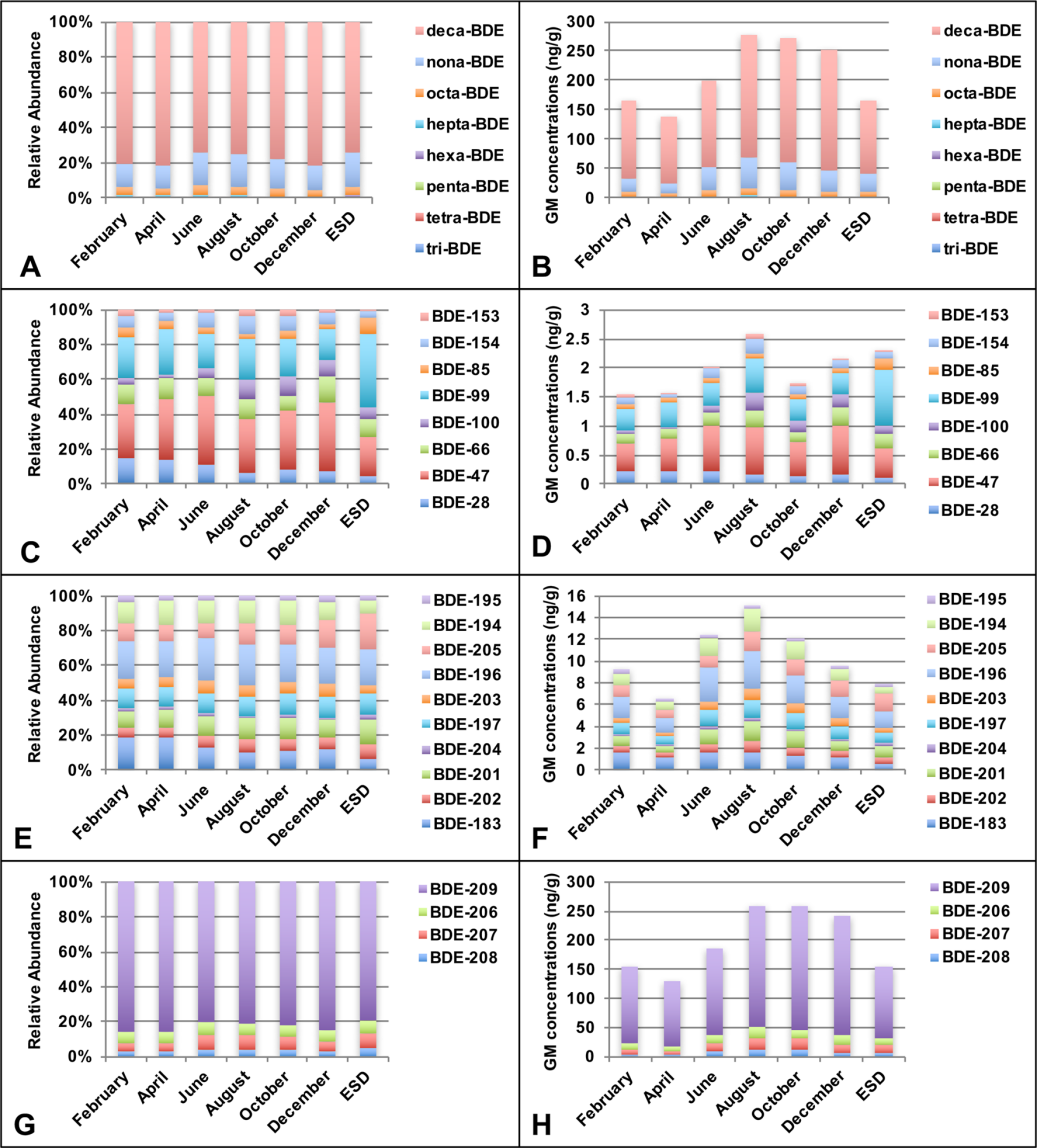
Relative abundance of tri-BDE to deca-BDE (**a**), BDE-28 to BDE-154 (**c**), BDE-183 to BDE-205 (**e**), BDE-206 to BDE-209 (**g**) and GM concentrations of tri-BDE to deca-BDE (**b**), BDE-28 to BDE-154 (**d**), BDE-183 to BDE-205 (**f**), BDE-206 to BDE-209 (**h**) in FD and ESD

The GM concentrations of total PBDEs every second month in FD were 167 ng/g (February), 139 ng/g (April), 200 ng/g (June), 278 ng/g (August), 272 ng/g (October), and 254 ng/g (December) (Fig. 1b). It is anticipated that PBDE levels from June to October (summer and early autumn) in FD exceeded those in other months since higher room temperature in warmer months may lead to more volatile emissions of PBDEs from sources like electrical appliances and furnishings, etc. (Zhang et al. 2009), but this is not wholly true. The temporal concentration variations of PBDEs between the maximum and minimum were approximately twofold, with the highest happened in August and lowest in April. For two main lower brominated congeners, levels of BDE-47 in FD showed tiny differences between months and were ranked as August (0.61, ng/g) ≈ June (0.55) ≈ December (0.52) ≈ October (0.46) > February (0.36) ≈ April (0.32), while the descending trend of BDE-99 was August (0.55, ng/g) ≈ June (0.53) > February (0.42) ≈ April (0.36) ≈ October (0.33) > December (0.25) (Fig. 1d). Increasing/decreasing emissions in warmer/colder months would probably be counterbalanced by indoor air conditioning and PBDEs levels exhibited generally temporal stability in Shanghai. The residents in Shanghai usually utilize air conditioners as heating system in winter (e.g., in February), and lowest room temperature of the year may occur in early spring. Thus, it can be deduced that room temperature may be another influencing factor of PBDEs levels in household indoor microenvironment, which is consistent with the ideas of Cao et al. (2014b) and Muenhor and Harrad (2012) .

With respect to congener profiles between months, there was a slight difference in FD (Fig. 1a). The levels of nona-BDE congeners from April to June increased sharply (Fig. 1b), but the percentage of sum concentrations of nona-BDEs and deca-BDE kept stable between two months, indicating that the increase of nona-BDEs levels may be due to the debromination of deca-BDE. Kajiwara et al. (2008) observed that BDE-209 partly debrominated to nona- and octa-BDE under natural sunlight conditions. Stapleton and Dodder (2008) also observed photolytic degradation of BDE-209 in house dust during the cumulative 200-h exposure to sunlight. For tri-BDEs to hexa-BDEs, there was a slight increase of sum concentrations from April to August (Fig. 1d), BDE-100 contributed to approximately 10% of ∑tri-hexa-BDEs from June to October, which was of small proportion in other months (Fig. 1c). It was assumed that higher room temperature would cause increased partitioning of low brominated PBDEs into the air. Besides, the ratio of BDE-47/BDE-99 was more than 1 every even month in FD while in ESD the ratio was 0.55 (possible reasons provided in “Comparison and relationship between FD and ESD” section). Figure 1f shows that the temporal fluctuation of concentrations of commercial octa-BDEs in FD from February to December was not statistically distinct. The proportion of BDE-183 was slightly higher in February and April. With respect to BDE-209, which contributed to about 80% of commercial deca-BDEs, dominated the total concentrations of PBDEs in FD every even month (Fig. 1g/h).

### Human exposure via dust ingestion

It is well known that exposure to environmental pollutants such as PBDEs will have an adverse impact on human health (Harrad et al. 2008). The human intake can occur from inhalation, dermal contact, and dust ingestion (Allen et al. 2008; Civan and Kara 2016). In domestic indoor microenvironment, dust ingestion is the main route of human exposure to PBDEs, particularly for toddlers who are deemed to be more susceptible (EPA 2008). In this study, our approach to estimate human exposure via dust ingestion took sample elevation into account, and we attempted to more scientifically assess risks for toddlers (1–3 years) and adults (≥ 18 years).

For exposure assessment, we followed the previous method (EPA 2008) with minor modifications. The estimated daily intake (EDI) of PBDEs via dust ingestion was calculated using the following equation (Wang et al. 2013; Abdallah and Covaci 2014):

$$ \mathrm{EDI}=\frac{IngR\times C\left(\mathrm{dust}\right)\times F}{BW} $$

Where IngR is the dust ingestion rate, g/day; *C* (dust) is the concentration of analyzed PBDEs in FD or ESD, ng/g; *F* is the percentage of daily time spent at home, %; and BW refers to body weight for toddlers and adults, kg.

Considering different regions have different human parameters, mean values of dust ingestion and body weight for toddlers in Shanghai were selected as 41 mg/day and 13.2 kg (MEPPRC 2016b), while for adults the mean values were 30 mg/day and 63.5 kg (MEPPRC 2016a). The corresponding time toddlers and adults spent indoors was assumed to be 1260 min/day (87.5%) and 1140 min/day (79.2%) (EPA 2008). Variable exposure scenarios were calculated by using 5th percentile, geometric mean, median, 95th percentile concentrations in FD and ESD.

EDI of PBDEs with different exposure scenarios for toddlers and adults is listed in Table 2. On the basis of GM concentration, ∑_22_ PBDEs exposure via mean dust ingestion estimated for toddlers in Shanghai were 0.55 ng/kg bw/day in FD and 0.45 ng/kg bw/day in ESD, and for adults 0.08 ng/kg bw/day in FD and 0.06 ng/kg bw/day in ESD, respectively. The highest daily intake (23.1 ng/kg bw/day) appeared in the group of toddlers via high dust ingestion at 95th percentile in FD, even twofold higher than that in ESD. However, using floor dust may overrate the PBDE exposure of adults who have less opportunity to contact with floor dust while toddlers have frequent hand-to-mouth contacts with floor dust. Elevated surface dust was assumed to be a preferable indicator of risk assessment for adults who were more vulnerable to contact with elevated house furnishings. For toddlers in Shanghai, the estimated exposure levels of BDE-47, BDE-99, BDE-153, and BDE-209 were by far 4739, 8772, 74,626, and 2933 times lower than their reference dose (RfD) values (Table S4).

**Table 2:** Estimated daily intake (EDI) of ∑PBDEs via dust ingestion for toddlers and adults, all values are in ng/kg bw/day

	Toddlers				Adults			
	5th	GM	Median	95th	5th	GM	Median	95th
FD (mean^a^ dust ingestion)	0.10	0.55	0.48	4.73	0.01	0.08	0.07	0.65
FD (high^b^ dust ingestion)	0.48	2.69	2.48	23.07	0.02	0.13	0.12	1.09
ESD (mean dust ingestion)	0.15	0.45	0.48	2.15	0.02	0.06	0.08	0.30
ESD (high dust ingestion)	0.73	2.19	2.98	10.5	0.03	0.10	0.14	0.49

^a^Mean dust ingestion rate for toddlers in Shanghai is 41 mg/day, for adults 30 mg/day

^b^High dust ingestion rate for toddlers is 200 mg/day, for adults 50 mg/day from Jones-Otazo et al. (2005)

Non-carcinogenic risk for main PBDE congeners (BDE-47, -99, -153, -209) was calculated by hazard quotient (HQ), and the results indicated that the non-cancer risk was so low as to be negligible (Table S5). Based on the information of the US EPA IRIS, BDE-209 is the only PBDEs associated with carcinogenic risk in human with neurobehavioral effects (EPA 2008; Civan and Kara 2016). The carcinogenic risk among toddlers and adults exposed to BDE-209 was evaluated and much lower than the threshold level (10^−6^) as shown in Table S6, which suggested the likelihood of low deleterious risk of BDE-209 exposure via dust ingestion in Shanghai. Our results were similar with studies in Belgium and UK (Ali et al. 2011a).

Estimating human exposure via dust ingestion only is influenced by many factors. For instance, the time people spend at home may be overestimated. In this study, we assumed 100% absorption efficiency of intake in accordance with most studies (Ali et al. 2011b; Hassan and Shoeib 2015). However, it is worthwhile noting that bioaccessibility of nearly all the PBDE congeners present in house dust may be less than 50% (Yu et al. 2012). Such assumption might still result in overestimation of human exposure to PBDEs. Unfortunately, there was no overall and authoritative dataset to record the bioaccessibility of PBDEs. More research needs to be done in the future concerning the bioaccessibility of PBDEs, to facilitate more reliable human health risk assessment.

## Conclusions

The residue levels of PBDEs in house dust (FD and ESD) from Shanghai were comparatively lower in comparison with that reported in North America and some European countries. In this study, total concentrations of 22 PBDE congeners in FD were mostly higher than those in ESD. BDE-209 was the predominant PBDE congener in both FD and ESD, suggesting that commercial deca-BDE products were its original sources. Concentrations of commercial penta-BDE compositions showed significant linear correlation between FD and ESD. No marked temporal variation was found in floor dust during the whole year with the relatively high level of PBDEs in August. By no-dietary ingestion, the estimated non-carcinogenic and carcinogenic risk levels of PBDEs for toddlers and adults were both below the EPA’s safe limits (1 and 10^−6^), indicating that people in this study have low-dose exposure risks when only neurobehavioral effects were considered. More works need to be done for human exposure to PBDEs in house dust via ingestion, inhalation, and dermal contact which would directly or indirectly affect the lifetime risk of cancer.

## Electronic supplementary material

ESM 1 (DOCX 1614 kb).
