# Supplementary material for: Occurrence of polybrominated diphenyl ethers in floor and elevated surface house dust from Shanghai, China
Source: Environ Sci Pollut Res Int. 2018 Apr 24;25(18):18049–58. doi: 10.1007/s11356-018-1968-4 (PMC6677682; doi:10.1007/s11356-018-1968-4)
Supplement: Supplementary file 1 — (DOCX 1614 kb). [file 11356_2018_1968_MOESM1_ESM.docx]

Supplementary Material

**Occurrence of polybrominated diphenyl ethers in floor and elevated surface house dust from Shanghai, China**

Dong Niu^1^, Yanling Qiu^1^, Li Li^1^, Yihui Zhou^2^, Xinyu Du^2^, Zhiliang Zhu^2^, Ling Chen^2^, Zhifen Lin^3^

^1^ Key laboratory of Yangtze River Water Environment, College of Environmental Science and Engineering, Tongji University, Shanghai, 200092

^2^ State Key Laboratory of Pollution Control and Resource Reuse, College of Environmental Science and Engineering, Tongji University, Shanghai, 200092

^3^ Shanghai Key Laboratory of Chemical Assessment and Sustainability, College of Environmental Science and Engineering, Tongji University, Shanghai, 200092

----------------------------------

Corresponding author：

Yanling Qiu

ylqiu@tongji.edu.cn

**Pages: 9, 6 Tables and 3 Figures.**

***Contents:***

**House Conditions**

**Table S1.** Limit of detection (LOD) and quantification (LOQ), dectection frequency for PBDE congeners in house dust samples.

**Table S2.** Median (or GM) concentrations of BDE-47, -99, -183, -209 (ng/g) in house dust measured in different studies.

**Table S3.** Paired-samples T test on concentrations of PBDE congeners between FD and ESD.

**Table S4.** Human exposure to BDE-47, -99, -153, -209 via dust ingestion for toddlers and adults. (×10^-5^ ng/kg bw/day.)

**Table S5.** Hazard Quotient (HQ) of BDE-47, -99, -153, -209 via dust ingestion for toddlers and adults.

**Table S6.** Cancer estimation of BDE-209 via dust ingestion exposure among toddlers and adults.

**Fig. S1** GM and average (arithmetic mean ± SD) concentrations of ∑tri-hepta-BDEs, ∑octa-BDEs, ∑nona-BDEs and deca-BDE (BDE-209) in two groups.

**Fig. S2** Profiles of tri-BDE to deca-BDE in FD and ESD.

**Fig. S3** Correlations between concentrations of three commercial BDEs in FD and ESD (n = 22).

**House Conditions**

Totally 22 houses in urban areas of Shanghai were investigated. A simple questionnaire, including the year of building, the latest time of decoration and the material of decoration, was required finished by family members. The use condition of main house appliances (e.g. television, air conditioner, refrigerator, computer, laundry machine) was also taken into consideration, and we found that most of house appliances were purchased after the year of 2007. TV, air conditioner, refrigerator were the three most frequently used. The average time of family members spent at home per day in each room was acquired in order to calculate human daily intake of PBDEs. Some diseases like respiratory disease were inquired in privacy.

**Table S1.** Limit of detection (LOD) and quantification (LOQ), dectection frequency for PBDE congeners in house dust samples.

| Congeners | LOD (ng/g) | LOQ (ng/g) | Detection frequency (FD) | | Detection frequency (ESD) |
| --- | --- | --- | --- | --- | --- |
| BDE-28 | 0.01 | 0.03 | | 97.6% | 72.2% |
| BDE-47 | 0.07 | 0.23 | | 98.4% | 83.3% |
| BDE-66 | 0.05 | 0.17 | | 92.8% | 83.3% |
| BDE-85 | 0.12 | 0.40 | | 21.6% | 50% |
| BDE-99 | 0.16 | 0.53 | | 80% | 100% |
| BDE-100 | 0.03 | 0.10 | | 66.4% | 72.2% |
| BDE-153 | 0.02 | 0.07 | | 36.8% | 94.4% |
| BDE-154 | 0.03 | 0.10 | | 80.8% | 61.1% |
| BDE-183 | 0.04 | 0.14 | | 100% | 100% |
| BDE-194 | 0.11 | 0.37 | | 99.2% | 83.3% |
| BDE-195 | 0.13 | 0.43 | | 70.4% | 66.7% |
| BDE-196 | 0.09 | 0.30 | | 99.2% | 100% |
| BDE-197 | 0.11 | 0.37 | | 99.2% | 100% |
| BDE-201 | 0.06 | 0.20 | | 100% | 100% |
| BDE-202 | 0.14 | 0.47 | | 92% | 100% |
| BDE-198,199,200,203 | 0.01 | 0.03 | | 100% | 88.9% |
| BDE-204 | 0.11 | 0.37 | | 68% | 66.7% |
| BDE-205 | 0.16 | 0.53 | | 96.8% | 100% |
| BDE-206 | 0.22 | 0.73 | | 100% | 100% |
| BDE-207 | 0.23 | 0.77 | | 100% | 100% |
| BDE-208 | 0.26 | 0.87 | | 100% | 100% |
| BDE-209 | 0.75 | 2.5 | | 100% | 100% |

**Table S2.** Median (or GM) concentrations of BDE-47, -99, -183, -209 (ng/g) in house dust measured in different studies.

| N | Country | Sampling year | BDE-47 | BDE-99 | BDE-183 | BDE-209 | Reference |
| --- | --- | --- | --- | --- | --- | --- | --- |
| 129 | Poland | 2012-2013 | <2 (3.80) | <2 (4.50) |  | 270 (345) | Korcz et al. (2017) |
| 28 | Portugal | 2010-2011 | 5.70 | 6.30 | 2.40 | 270 | Coelho et al. (2016) |
| 40 | Turkey | 2015-2016 | 10.0 | 6.45 | 20.5 | 138 | Civan and Kara (2016) |
| 17 | Egypt | 2013 | 1.70 | 2.70 | 1.10 | 40.2 | Hassan and Shoeib (2015) |
| 30 | USA | 2012 | (452) | (741) |  | (1720) | Stapleton et al. (2014) |
| 31 | South Africa | 2010-2011 | 2.60 (1.32) | 2.60 (1.84) |  | <1.8 (3.47) | Kefeni et al. (2014) |
| 43 | Belgium | 2008 | 8 (8) | 9 (11) | 2 (2) | 317 (306) | Ali et al. (2011) |
| 33 | New Zealand |  | 24.2 | 31.5 | 2.7 | 598 | Coakley et al. (2013) |
| 10 | Sweden |  | 42 | 52 | 12 | 320 | Thuresson et al. (2012) |
| 10 | UK | 2013-2014 | 11.5 | 20.4 | 10.3 | 2660 | Al-Omran and Harrad (2016) |
| 13 | Korea | 2009 |  |  |  | 829 | Kim et al. (2016) |
| 10 | Hangzhou, China | 2013 | 4.0 (5.2) | 3.6 (4.0) | 1.5 (1.2) | 228 (227) | Sun et al. (2016) |
| 216 | Nanjing, China | 2011 | 8.41 (6.55) | 1.69 (1.72) | 4.01 (3.65) | 63.2 (68.3) | Wang et al. (2015) |
| 132 | Shanghai, China (FD) | 2016 | 0.48 (0.65) | 0.39 (0.41) | 1.34 (1.45) | 139 (160) | This study |
| 22 | Shanghai,  China (ESD) | 2016 | 0.57 (0.53) | 0.91 (0.95) | 0.82 (0.53) | 160 (123) | This study |

**Table S3** Paired-samples T test on concentrations of PBDE congeners between FD and ESD.

| **Congeners** | ***P*** |
| --- | --- |
| BDE-28 | 0.2055 |
| BDE-47 | 0.3645 |
| BDE-66 | 0.2147 |
| BDE-85 | 0.0106 |
| BDE-99 | 0.3029 |
| BDE-100 | 0.7362 |
| BDE-153 | 0.0007 |
| BDE-154 | 0.5370 |
| BDE-183 | 0.0064 |
| BDE-194 | 0.1741 |
| BDE-195 | 0.1657 |
| BDE-196 | 0.2140 |
| BDE-197 | 0.2050 |
| BDE-201 | 0.3168 |
| BDE-202 | 0.3426 |
| BDE-198, -199, -200, -203 | 0.2416 |
| BDE-204 | 0.6774 |
| BDE-205 | 0.4089 |
| BDE-206 | 0.2015 |
| BDE-207 | 0.2802 |
| BDE-208 | 0.2101 |
| BDE-209 | 0.0440 |

**Table S4.** Estimated daily intake (EDI) of BDE-47, -99, -153, -209 via dust ingestion for toddlers and adults. (×10^-5^ ng/kg bw/day.)

|  |  |  | RfD | 5^th^ | GM | Median | 95^th^ |
| --- | --- | --- | --- | --- | --- | --- | --- |
| FD | Toddlers | BDE-47 | 1×10^7^ | 39.9 | 176 | 130 | 2110 |
|  |  | BDE-99 | 1×10^7^ | 21.7 | 111 | 106 | 1140 |
|  |  | BDE-153 | 2×10^7^ | 2.71 | 10.9 | 2.71 | 268 |
|  |  | BDE-209 | 7×10^8^ | 6140 | 43400 | 37700 | 362000 |
|  | Adults | BDE-47 | 1×10^7^ | 5.49 | 24.3 | 18.0 | 292 |
|  |  | BDE-99 | 1×10^7^ | 2.99 | 15.3 | 14.6 | 157 |
|  |  | BDE-153 | 2×10^7^ | 0.374 | 1.50 | 0.374 | 36.9 |
|  |  | BDE-209 | 7×10^8^ | 847 | 5990 | 5200 | 49900 |
| ESD | Toddlers | BDE-47 | 1×10^7^ | 9.50 | 144 | 155 | 2010 |
|  |  | BDE-99 | 1×10^7^ | 63.3 | 261 | 247 | 2690 |
|  |  | BDE-153 | 2×10^7^ | 2.71 | 2.71 | 2.71 | 138 |
|  |  | BDE-209 | 7×10^8^ | 7620 | 33400 | 43400 | 170000 |
|  | Adults | BDE-47 | 1×10^7^ | 1.31 | 19.8 | 21.3 | 278 |
|  |  | BDE-99 | 1×10^7^ | 8.72 | 35.9 | 34.0 | 371 |
|  |  | BDE-153 | 2×10^7^ | 0.374 | 0.374 | 0.374 | 19.1 |
|  |  | BDE-209 | 7×10^8^ | 1050 | 4600 | 5990 | 23700 |

**Table S5** Hazard Quotient (HQ) of BDE-47, -99, -153, -209 via dust ingestion for toddlers and adults.

|  |  |  | HQ (5^th^) | HQ (GM) | HQ (Median) | HQ (95^th^) |
| --- | --- | --- | --- | --- | --- | --- |
| FD | Toddlers | BDE-47 | 3.99×10^-11^ | 1.76×10^-10^ | 1.30×10^-10^ | 2.11×10^-9^ |
|  |  | BDE-99 | 2.17×10^-11^ | 1.11×10^-10^ | 1.06×10^-10^ | 1.14×10^-9^ |
|  |  | BDE-153 | 1.36×10^-12^ | 5.45×10^-12^ | 1.36×10^-12^ | 1.34×10^-10^ |
|  |  | BDE-209 | 8.77×10^-11^ | 6.20×10^-10^ | 5.39×10^-10^ | 5.17×10^-9^ |
|  | Adults | BDE-47 | 5.49×10^-12^ | 2.43×10^-11^ | 1.80×10^-11^ | 2.92×10^-10^ |
|  |  | BDE-99 | 2.99×10^-12^ | 1.53×10^-11^ | 1.46×10^-11^ | 1.57×10^-10^ |
|  |  | BDE-153 | 1.87×10^-13^ | 7.50×10^-13^ | 1.87×10^-13^ | 1.85×10^-11^ |
|  |  | BDE-209 | 1.21×10^-11^ | 8.56×10^-11^ | 7.43×10^-11^ | 7.13×10^-10^ |
| ESD | Toddlers | BDE-47 | 9.50×10^-12^ | 1.44×10^-10^ | 1.55×10^-10^ | 2.01×10^-9^ |
|  |  | BDE-99 | 6.33×10^-11^ | 2.61×10^-10^ | 2.47×10^-10^ | 2.69×10^-9^ |
|  |  | BDE-153 | 1.36×10^-12^ | 1.36×10^-12^ | 1.36×10^-12^ | 6.9×10^-11^ |
|  |  | BDE-209 | 1.09×10^-10^ | 4.77×10^-10^ | 6.20×10^-10^ | 2.43×10^-9^ |
|  | Adults | BDE-47 | 1.31×10^-12^ | 1.98×10^-11^ | 2.13×10^-11^ | 2.78×10^-10^ |
|  |  | BDE-99 | 8.72×10^-12^ | 3.59×10^-11^ | 3.40×10^-11^ | 3.71×10^-10^ |
|  |  | BDE-153 | 1.87×10^-13^ | 1.87×10^-13^ | 1.87×10^-13^ | 9.55×10^-12^ |
|  |  | BDE-209 | 1.50×10^-11^ | 6.57×10^-11^ | 8.56×10^-11^ | 3.39×10^-10^ |

**Table S6** Cancer estimation of BDE-209 via dust ingestion exposure among toddlers and adults.

|  |  | R (5^th^) | R (GM) | R (Median) | R (95^th^) |
| --- | --- | --- | --- | --- | --- |
| FD | Toddlers | 4.30×10^-8^ | 3.04×10^-7^ | 2.64×10^-7^ | 2.53×10^-6^ |
|  | Adults | 5.93×10^-9^ | 4.19×10^-8^ | 3.64×10^-8^ | 3.49×10^-7^ |
| ESD | Toddlers | 5.33×10^-8^ | 2.34×10^-7^ | 3.04×10^-7^ | 1.19×10^-6^ |
|  | Adults | 7.35×10^-9^ | 3.22×10^-8^ | 4.19×10^-8^ | 1.66×10^-7^ |


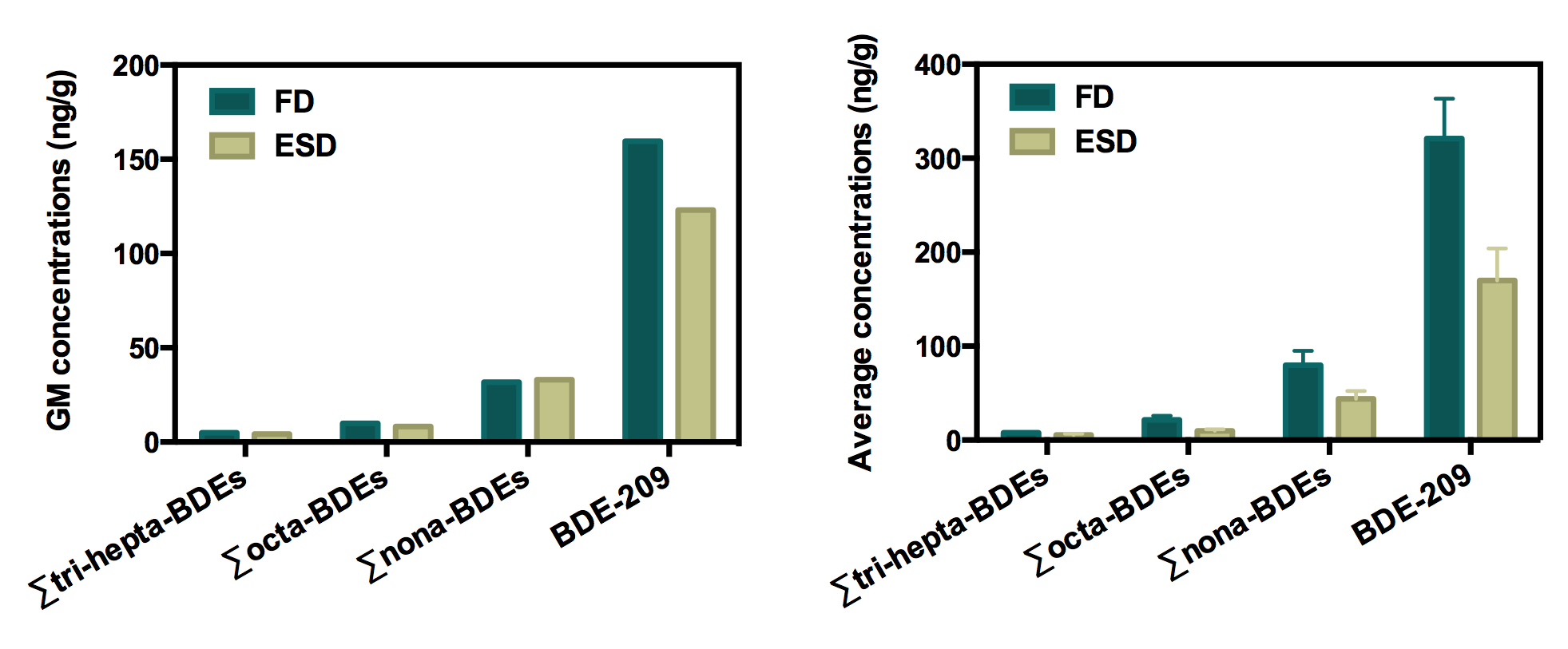
**Fig. S1** GM and average (arithmetic mean ± SD) concentrations of ∑tri-hepta-BDEs, ∑octa-BDEs, ∑nona-BDEs and deca-BDE (BDE-209) in two groups.





**Fig. S2** Profile of tri-BDE to deca-BDE in FD and ESD.


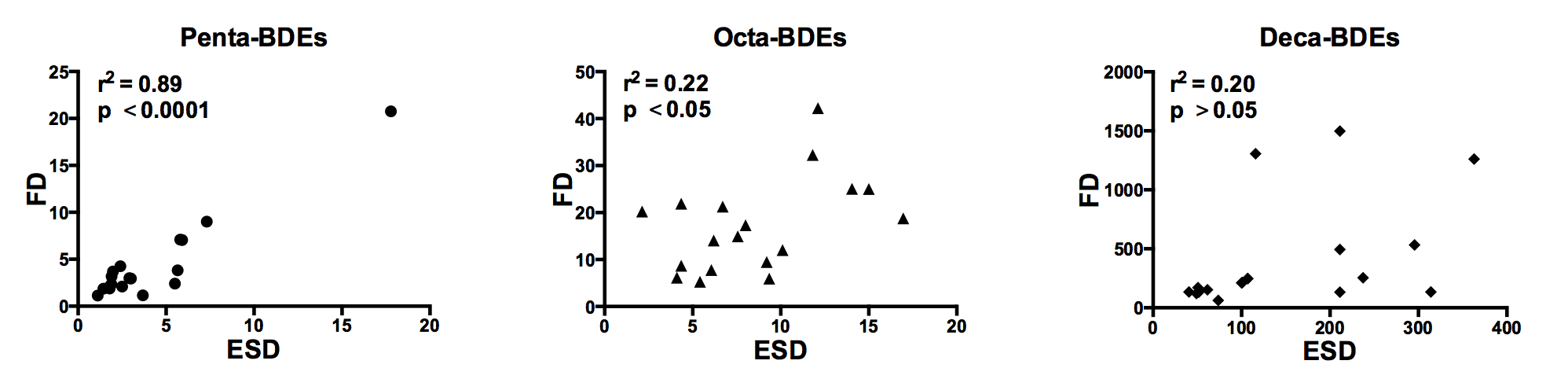


**Fig. S3** Correlations between concentrations of three commercial BDEs in FD and ESD (n = 22).

**References:**

Al-Omran LS, Harrad S (2016) Distribution pattern of legacy and "novel" brominated flame retardants in different particle size fractions of indoor dust in Birmingham, United Kingdom. Chemosphere 157: 124-131.

Ali N, Harrad S, Goosey E, Neels H, Covaci A (2011) "Novel" brominated flame retardants in Belgian and UK indoor dust: Implications for human exposure. Chemosphere 83: 1360-1365.

Civan MY, Kara UM (2016) Risk assessment of PBDEs and PAHs in house dust in Kocaeli, Turkey: levels and sources. Environ Sci Pollut Res Int.

Coakley JD, Harrad SJ, Goosey E, Ali N, Dirtu AC, Van den Eede N, Covaci A, Douwes J, 't Mannetje A (2013) Concentrations of polybrominated diphenyl ethers in matched samples of indoor dust and breast milk in New Zealand. Environ Int 59: 255-261.

Coelho SD, Sousa ACA, Isobe T, Kim JW, Kunisue T, Nogueira AJA, Tanabe S (2016) Brominated, chlorinated and phosphate organic contaminants in house dust from Portugal. Sci Total Environ 569: 442-449.

Hassan Y, Shoeib T (2015) Levels of polybrominated diphenyl ethers and novel flame retardants in microenvironment dust from Egypt: An assessment of human exposure. Sci Total Environ 505: 47-55.

Kefeni KK, Okonkwo JO, Botha BM (2014) Concentrations of polybromobiphenyls and polybromodiphenyl ethers in home dust: Relevance to socio-economic status and human exposure rate. Sci Total Environ 470: 1250-1256.

Kim SK, Kim KS, Hong S (2016) Overview on relative importance of house dust ingestion in human exposure to polybrominated diphenyl ethers (PBDEs): International comparison and Korea as a case. Sci Total Environ 571: 82-91.

Korcz W, Strucinski P, Goralczyk K, Hernik A, Lyczewska M, Matuszak M, Czaja K, Minorczyk M, Ludwicki JK (2017) Levels of polybrominated diphenyl ethers in house dust in Central Poland. Indoor Air 27: 128-135.

Stapleton HM, Misenheimer J, Hoffman K, Webster TF (2014) Flame retardant associations between children's handwipes and house dust. Chemosphere 116: 54-60.

Sun J, Wang Q, Zhuang S, Zhang A (2016) Occurrence of polybrominated diphenyl ethers in indoor air and dust in Hangzhou, China: Level, role of electric appliances, and human exposure. Environ Pollut 218: 942-949.

Thuresson K, Bjorklund JA, de Wit CA (2012) Tri-decabrominated diphenyl ethers and hexabromocyclododecane in indoor air and dust from Stockholm microenvironments 1: Levels and profiles. Sci Total Environ 414: 713-721.

Wang BL, Pang ST, Zhang XL, Li XL, Sun YG, Lu XM, Zhang Q (2015) Levels of Polybrominated Diphenyl Ethers in Settled House Dust From Urban Dwellings With Resident Preschool-Aged Children in Nanjing, China. Arch Environ Con Tox 68: 9-19.
